# Supplementary material for: A single-dose live-attenuated vaccine prevents Zika virus pregnancy transmission and testis damage
Source: Nat Commun. 2017 Sep 22;8:676. doi: 10.1038/s41467-017-00737-8 (PMC5610254; doi:10.1038/s41467-017-00737-8)
Supplement: Supplementary file 1 — Supplementary Information [file 41467_2017_737_MOESM1_ESM.pdf]

File name: Supplementary Information
Description: Supplementary Figures and Supplementary Table

File name: Peer Review File

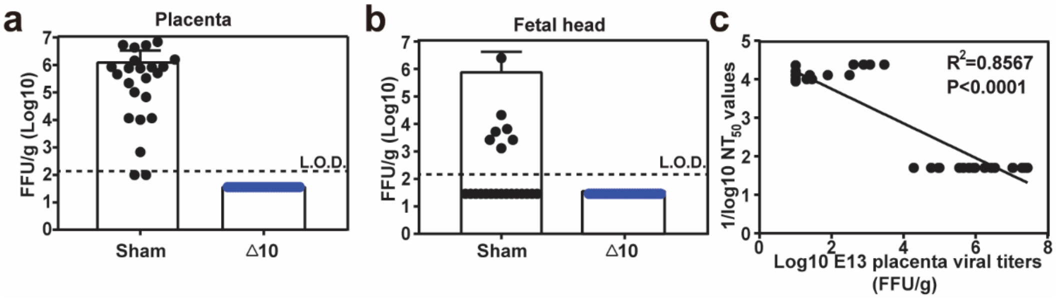

Supplementary Figure 1. **Infectious ZIKV burden in placentas and fetal heads from sham or ZIKV-3'UTR-Δ10-LAV-immunized dams**. In the pregnancy protection experiment (see details in **Fig 1**), at day 7 post-challenge (equivalent to E13), placenta (**a**) and fetal heads (**b**) were collected from PBS sham and ZIKV-3'UTR-Δ10-LAV-immunized dams, and quantified for infectious ZIKV using a focus forming assay. Dashed lines indicate limit of detection (L.O.D.) of the assays. Results are pooled from two independent biological experiments, and each symbol represents data from an individual placenta (n = 23) or fetus (n = 30). (**c**) Correlation of E13 placenta viral burden with antibody neutralizing NT_{50} values of ZIKV-3'UTR-Δ10-LAV. *P* and R^2 values reflect Pearson correlation tests. All negative samples are plotted at the half value of L.O.D. Error bars represent standard deviations.

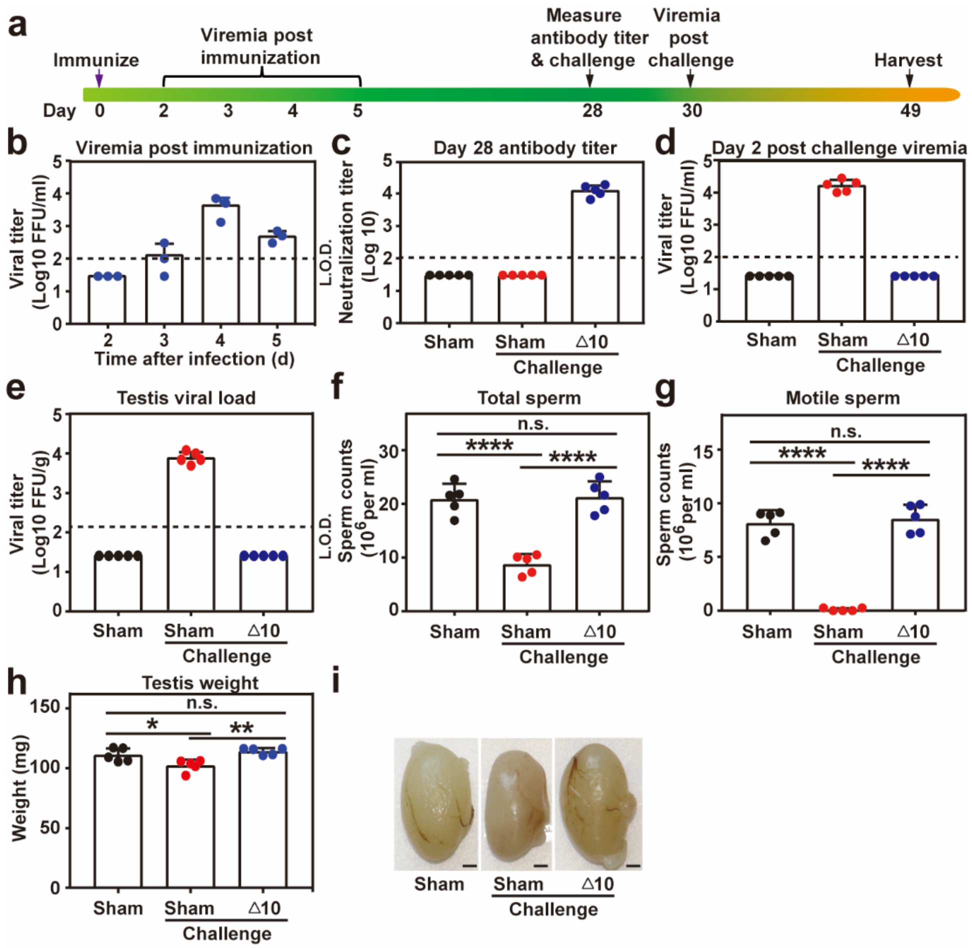

Supplementary Figure 2. **ZIKV-3'UTR-Δ10-LAV protects adult A129 male mice against testis infection and injury**. (**a**) Scheme of immunization of 15-week-old A129 male mice with 1×10^4 FFU of ZIKV-3'UTR-Δ10-LAV (Δ10; n = 5) or PBS sham (n = 5). At day 28 post-immunization, mice were measured for neutralizing antibody titers. On the same day, the mice were challenged with 10^6 FFU of ZIKV-PRVABC59. Peak viremia was measured at day 2 post-challenge (day 30 post-immunization). At day 49 post-immunization, mice were euthanized and measured for total and motile sperm counts and viral loads in the testis. (**b**) Viremia after ZIKV-3'UTR-Δ10-LAV immunization. (**c**) NT_{50} values of antibody neutralization at day 28 post-immunization. Antibody neutralizing titers were measured for individual animals in each group by an mCherry ZIKV. The dashed lines indicate the limit of detection (L.O.D.) of the assay. (**d**) Day 2 post-challenge (day 30 post-immunization) viremia. At day 21 post-challenge, animals from each group were analyzed for testis viral load (**e**), total sperm counts (**f**), motile sperm counts (**g**), testis weight (**h**), and testis size (**i**). Representative images of testis are presented in (**i**). Scale bar, 1 mm. Asterisks indicate significant differences (One-way ANOVA: *, *P* value < 0.05; **, *P* value < 0.01; ****, *P* value < 0.0001). Non-significant (n.s.), *P* value > 0.5. All negative samples are plotted at the half value of L.O.D. Error bars represent standard deviations.

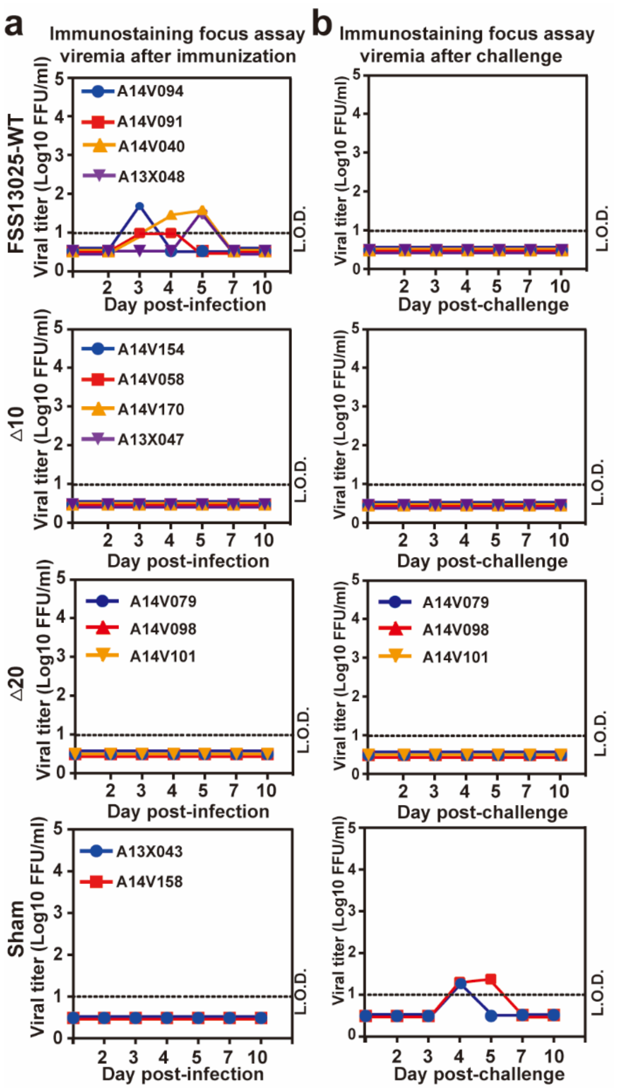

Supplementary Figure 3. Infectious virus in serum of challenged rhesus macaque. Infectious virus in RM serum (viremia) collected at days 2, 3, 4, 5, 7, and 10 post-immunization (**a**) or post-challenge (**b**) was quantified by a focus forming assay. See detailed experimental scheme in **Fig 3**. Dashed lines indicate limit of detection (L.O.D.) of the assays. All negative samples are plotted at the half value of L.O.D.

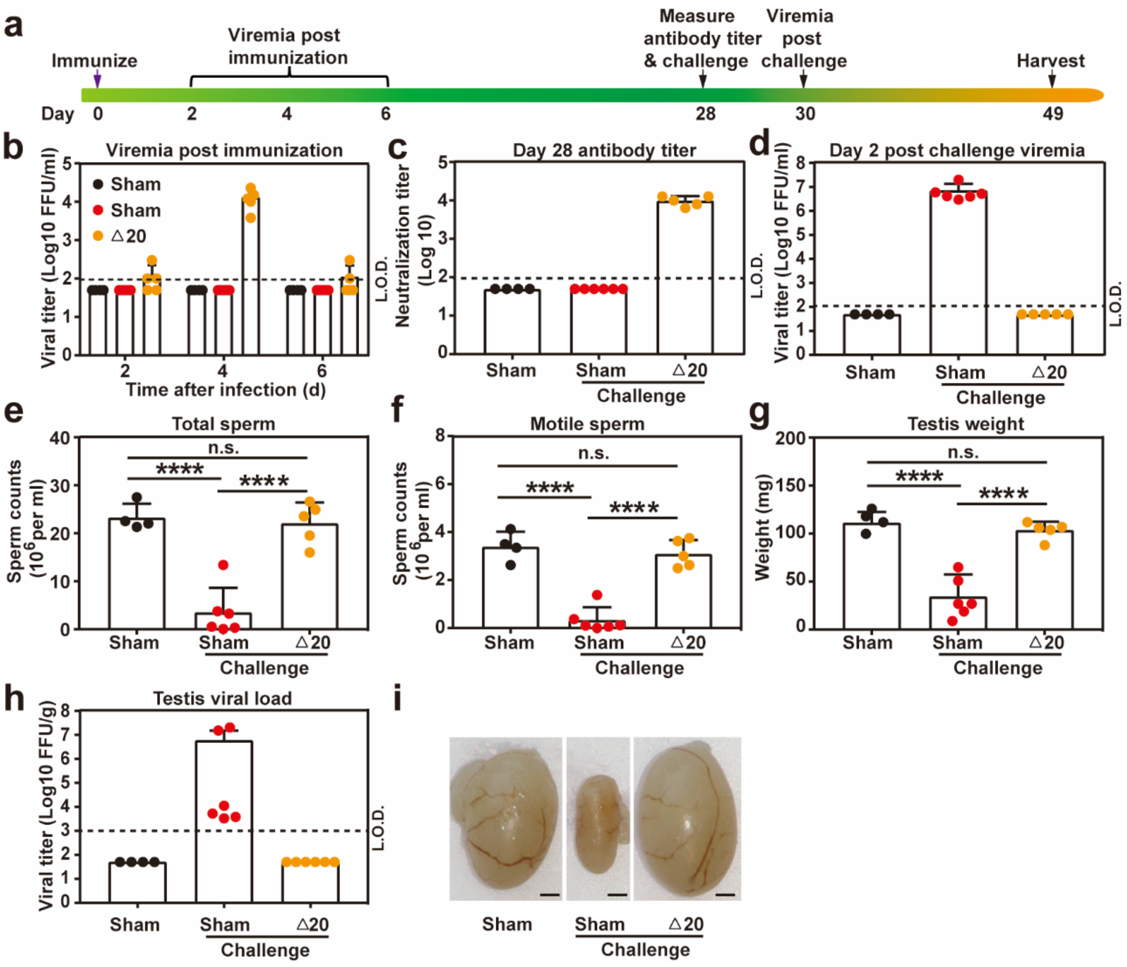

Supplementary Figure 4. ZIKV-3'-UTR-Δ20-LAV protects young A129 male mice against testis infection and injury. (**a**) Scheme of immunization of 3-week-old A129 male mice with 10^3 FFU of ZIKV-3'-UTR-Δ20-LAV (Δ20; n = 6) or PBS sham (n = 4 or 6). At day 28 post-immunization, immunized mice were measured for neutralization antibody titers. At the same day, mice from one sham group and mice from Δ20-immunized group were challenged with 10^6 FFU of ZIKV-PRVABC59. Viremia was measured at day 2 post-challenge (day 30 post-immunization). At day 49 post-immunization, mice were analyzed for sperm counts and viral loads in testis. (**c**) NT_{50} values of antibody neutralization at day 28 post-immunization were measured for individual animals in each group. The dashed lines indicate the limit of detection (L.O.D.) of the assay. (**d**) Day 2 post-challenge (day 30 post-immunization) viremia. (**e-f**) Total (**e**) and motile (**f**) sperm counts at day 21 post-challenge (equivalent to day 49 post-immunization). (**g**) Testis weight from animals from sham, sham with challenge, and Δ20-immunized and challenged groups at day 21 post-challenge. (**h**) Viral load in testis at day 21 post-challenge. (**i**) Representative images of testis harvested at day 21 post-challenge. Scale bar, 1 mm. Asterisks indicate significant differences (One-way ANOVA: ****, *P* value < 0.0001). Non-significant (n.s.) with *P* value > 0.5. All negative samples are plotted at the half value of L.O.D. Error bars represent standard deviations.

Sequencing results for ZIKV-3'UTR-Δ20-LAV P5 viruses

	E	NS1
Selection I	T315I	-
Selection II	K443N	W98L
Selection III	K443N	-

Supplementary Table 1. Stability analysis of ZIKV-3'UTR-Δ20-LAV in cell culture. P0 viruses (derived from the culture fluids of RNA-transfected cells) were continuously cultured on Vero cells for five rounds (5 days for each round of culture), resulting in P5 viruses. The complete genomes of P5 mutant viruses were sequenced. All P5 viruses retained the 20-nucleotide deletion in the 3'UTR. In addition, several adaptive mutations are recovered; these mutations are presented by their amino acid positions of indicated genes based on ZIKV FSS13025 strain (GenBank number KU955593.1). Results from three independent passages (selections I, II, and II) are presented.
